# Incomplete Reporting of Baseline Characteristics in Clinical Trials: An Analysis of Randomized Controlled Trials and Systematic Reviews Involving Patients with Chronic Low Back Pain

**DOI:** 10.1371/journal.pone.0058512

**Published:** 2013-03-07

**Authors:** Maria M. Wertli, Manuela Schöb, Florian Brunner, Johann Steurer

**Affiliations:** 1 Horten Center for Patient Oriented Research and Knowledge Transfer, Department of Internal Medicine, University of Zurich, Zurich, Switzerland; 2 Department of Physical Medicine and Rheumatology, Balgrist University Hospital, Zurich, Switzerland; University of Louisville, United States of America

## Abstract

**Objective:**

The aim of this study was to evaluate the reporting of relevant prognostic information in a sample of randomized controlled trials (RCTs) that investigated treatments for patients with chronic low back pain (LBP). We also analysed how researchers conducting the meta-analyses and systematic reviews addressed the reporting of relevant prognostic information in RCTs.

**Methods:**

We searched the Cochrane Database to identify systematic reviews that investigated non-surgical treatments for patients with chronic LBP. The reported prognostic information was then extracted from the RCTs included in the reviews. We used a purpose-defined score to assess the quantity of information reported in the RCTs. We also determined how the authors of systematic reviews addressed the question of comparability of patient populations between RCTs.

**Results:**

Six systematic reviews met our inclusion criteria, and we analysed 84 RCTs. Based on the scores, the reporting of important prognostic variables was incomplete in almost half of the 84 RCTs. Information regarding patients’ general health, social support, and work-related conditions was rarely reported. Almost half of the studies included in one of the meta-analyses provided insufficient information that did not allow us to determine whether patients in the primary trials were comparable.

**Conclusions:**

Missing prognostic information potentially threatens the external validity (i.e. the generalizability or applicability) not only of primary studies but also of systematic reviews that investigate treatments for LBP. A detailed description of baseline patient characteristics that includes prognostic information is needed in all RCTs to ensure that clinicians can determine the applicability of the study or review results to their patients.

## Introduction

Assessing the external validity of randomized controlled trials (RCTs) is a key step in the critical appraisal of clinical studies. Many clinicians trust authors and journal editors to verify the high internal validity of the published studies (e.g., concealment of randomization list, information about drop-outs, intention to treat analysis), but physicians must decide for themselves whether the results apply to an individual patient. The information that is needed for this determination is reported in the Methods and Results sections of journal articles. The Methods section reports the eligibility criteria information, which states the patient qualifications for inclusion in the study. Patient characteristics are reported in the Results section; quite often, the article’s Table 1 shows the distribution of characteristics of patients included in the study. Guidelines for reporting, e.g., the CONSORT Statement for randomized controlled trials [1], recommend not only a comprehensive description of eligibility criteria but also a list of baseline characteristics for important prognostic factors.

**Table 1:** Important prognostic risk factor domains and subdomains in patients with low back pain (modified from Hayden et al. [7]).

Domain	Subdomain	SQR Score*
General patient characteristics	Socio-demographic status Social support	Minimal requirement: ≥1 subdomain reported
Baseline health status	Overall health Overall psychological health Previous LBP	Minimal requirement: ≥1 subdomain reported
Work-related factors	Work: psychosocial demands Work: physical demands Work history Work place attributes	Minimal requirement: ≥1 subdomain reported
Current LBP	Clinical history Disability related to the complaint Changes related to complaint over time	Minimal requirement: ≥1 subdomain reported
Clinical examination findings	Physical examination findings Definition of NSLBP diagnosis† Changes found during the physical exam	Minimal requirement: ≥1 subdomain reported
Interactions with work/society	Compensation issues related to LBP	Minimal requirement: ≥1 subdomain reported

LBP: low back pain; NSLBP: nonspecific low back pain;

† To fulfil this subdomain, at least one more attribute (in addition to pain duration) had to be reported (e.g. disability, severity, pain referral) [30];

* SQR: Score for the quantity of reporting: Scoring SQR high: information reported in one or more subdomains for all six main domains; SQR moderate: information reported in one or more subdomains for five main domains; SQR low: information reported in one or more subdomains for four or fewer main domains.

A complete description of relevant prognostic factors is particularly important in otherwise ill-defined diseases, such as chronic low back pain (CLBP). Several prognostic factors have been identified that can affect treatment effects in patients with CLBP, including age, duration of symptoms, first or recurrent episode, employment status, and comorbidities such as depression [2], [3]. For example, a treatment is effective in patients without depression but be less effective or even ineffective in depressed patients [4].

Knowing the patients’ baseline characteristics is important for interpreting study results, both for clinicians and for the researchers who conduct systematic reviews and meta-analyses. Pooling the results of primary studies with unknown or different distributions of relevant prognostic factors in the included population may lead to a biased result [5]. It is unclear whether authors report important prognostic information in sufficient detail in primary studies so as to be helpful in rational pooling of data in meta-analyses and systematic reviews.

The aim of the current study was to evaluate the reporting of relevant prognostic information in a selection of randomized controlled trials (RCTs) investigating treatment outcomes in patients with CLBP. We also determined whether the authors of systematic reviews addressed the question of comparability of patient populations between RCTs.

## Methods

### Study Design

Here we analysed primary studies included in CLBP-related systematic reviews in the Cochrane library. For the purpose of the current study, CLBP represents an ill-defined disease with high health care expenditure [6] for which important prognostic information is known to influence the course of the disease [3], [7]. We aimed to include a complete set of trials for each treatment intervention; therefore, we analysed primary studies that were included in systematic reviews published in the Cochrane library. The Cochrane Collaboration Guideline [8], [9], [10] has published guidelines for the standardized assessment of baseline characteristics to facilitate comparison of systematic reviews. While this study is not a systematic review reporting will be based, if applicable, on the recommendations of the PRISMA statement [11].

### Eligibility Criteria and Selection of Systematic Reviews

All systematic reviews that were published in the Cochrane library from its inception (1996) to December 2010 that investigated non-surgical treatments for CLBP were eligible for inclusion in our analysis. We searched the Cochrane library for the terms “chronic” and “non-specific low back pain” in the title, abstract, or keywords. Of the returned reviews, only RCTs published in English and German were eligible for further analysis due to the authors’ lack of proficiency in other languages. Non-randomized trials and observational studies were excluded.

Two reviewers (MW and MS) independently screened the titles and abstracts of the identified systematic reviews to determine which ones met the pre-defined inclusion criteria. The full text of each RCT included in the systematic reviews were then independently reviewed (MW and MS). Discrepancies between the two reviewers were discussed and resolved by consensus or by a third party (FB).

### Data Extraction and Synthesis

One reviewer (MS) extracted data from the RCTs, including bibliographic data (authors, year of publication), eligibility criteria, and prognostic information. Prognostic information for LBP was defined a priori in collaboration with experienced clinicians (one internist, one rheumatologist, one general practitioner) and one methodologist in the field and by consulting the relevant literature [2], [3].

We used the prognostic domains proposed by Hayden et al. [7] to categorize the information reported in the RCTs. These domains, which are considered to represent clinically meaningful groups, [2] have been used in previous research and are based on expert consensus [12]. The following six main domains were used: general patient characteristics, baseline health status, work-related factors, current low back pain (LBP), clinical examination findings, and interactions with work/society. Each main domain is divided into subdomains (e.g., current LBP is further divided according to the patient’s clinical history, disability related to the complaint, and changes in the complaint over time). There were a total of 16 prognostic subdomains (Table 1). The six main domains represent a spectrum of important information that helps clinicians decide whether the study results are applicable to their patients.

One reviewer (MW) confirmed all of the extracted information and assigned the data to the appropriate subdomains. To quantify the amount of reported prognostic information for each RCT, we defined a Score for the Quantity of Reporting (SQR) for each one as follows: High SQR, information was reported for one or more subdomain in all six main domains; moderate SQR, information was reported for one or more subdomains in five of the six main domains; and low SQR, information was reported for one or more subdomains in four or fewer main domains (Table 1).

The SQR for each study was then compared to how the baseline characteristics were assessed in the systematic reviews. Assessment of the comparability of baseline characteristics in studies is defined in the Method guidelines for systematic reviews in the Cochrane Collaboration Back Review Group for Spinal Disorders [8] (first published in 1997). The relevant question is: “Are the baseline characteristics similar with regards to the most important prognostic factors?” The possible answers are “Yes/No/Don’t know,” and studies were divided into “Yes”, “No”, or “Can’t tell” categories depending on the answer to that question. The updated Method Guidelines in 2003 [9] further stated, “In order to qualify for a “Yes,” groups have to be similar at baseline regarding demographic factors, duration and severity of complaints, percentage of patients with neurologic symptoms.” When not enough information is reported, the study must be classified as “Can’t tell”. We would expect that for primary studies with low SQRs, the answer to the above question would be “Can’t tell.” We also investigated whether studies with low SQR or that were classified as “Can’t tell” were included in the meta-analysis.

### Statistical Analysis

Descriptive statistics were used to summarize findings across the entire set of RCTs. We wished to evaluate changes in the quantity of reporting over time, particularly after the publication of the CONSORT statement in 1996 [1], which aimed to improve the quality of reporting in RCTs. Toward this end, the mean number of reported subdomains before and after 1998 (to allow one year for implementation of CONSORT suggestions) was compared using the t-test. Analyses were conducted with SPSS for Windows version 19 (IBM SPSS; Chicago, IL USA) and R statistical software for Windows (http://www.R-project.org/).

### Ethics Statement

For this study no ethical approval was required. No protocol was published or registered. All methods were determined a priori.

## Results

### Study Selection

**Figure 1:**
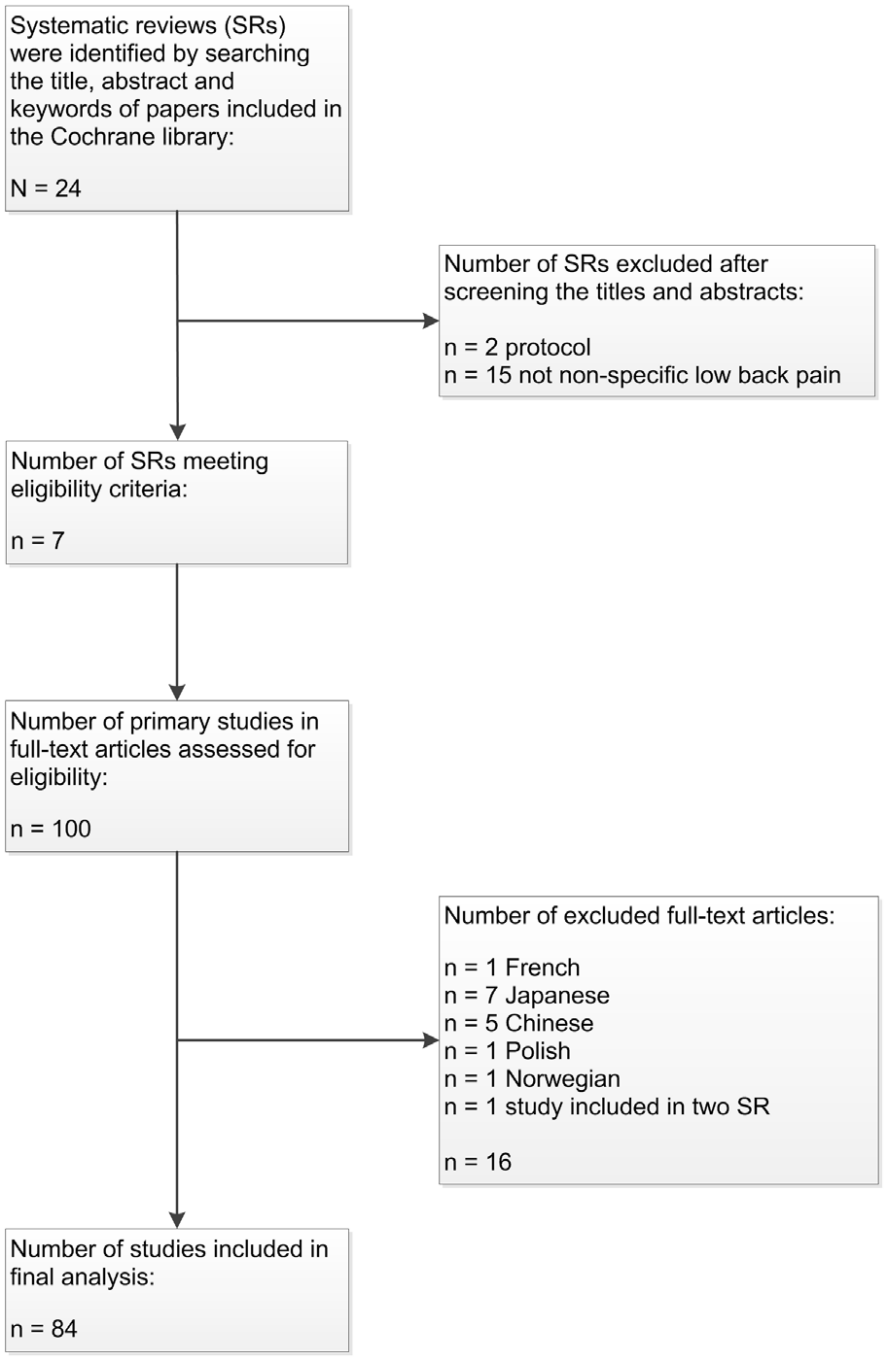
Study flow chart. The Cochrane Database of Systematic Reviews was searched in November, 2010.

Seven systematic reviews met the eligibility criteria (Figure 1). The reviews were published between 2005 and 2010 and included 100 primary studies. A total of 84 primary studies (RCTs) were included in the analysis. The main reason for exclusion was publication in a language other than English (n = 16). Figure 1 shows a flow diagram of the study selection process.

### Study Characteristics

Table 2 summarizes the objectives, the number of included RCTs, and the conclusions of each systematic review. Most RCTs aimed to investigate treatments only for chronic low back pain; few studies included patients with subacute and acute low back pain. The number of RCTs included in each systematic review ranged from four [13] to thirty-two [14] trials. The RCTs were published between 1971 and 2009. More than half of the studies assessed the effects of acupuncture (n = 18, 21.4%) or cognitive behavioural therapy (n = 27, 32.1%). Most patients in the control groups received placebo (n = 26, 30%), sham procedures (n = 12, 14%), or usual care (n = 12, 14%), or the patients were placed on a waiting list (n = 13, 15%). In most studies, the follow-up time was about 6 months (median 6 months, range 1 hour to 5 years). Details are shown in Table 3.

**Table 2:** Summaries of the systematic reviews in our analysis.

Author	Year	Objective	Number of studiesanalysed		Conclusion
Furlan et al. [32]	2005	To assess the effects of AC for the treatment of NSLBP and the effects of dry-needling for myofascial pain syndrome in the low-back region.		20	Acute LBP: no firm conclusions about the effectiveness of AC. Chronic LBP: AC more effective for pain relief and functional improvement than no treatment or sham treatment and in the short-term only. AC is not more effective than other conventional treatments.
Urquhart et al. [33]	2008	To determine whether antidepressants are more effective than placebo for the treatment of NSLBP		9	No clear evidence that antidepressants are more effective than placebo in the management of patients with CLBP.
Henschke et al. [14]	2010	To determine the effects of behavioural therapy for CLBP and the most effective behavioural approach		32	Short-term: moderate quality evidence that operant therapy is more effective than being placed on a waiting list and that behavioural therapy is more effective than usual care for pain relief. No specific type of behavioural therapy is more effective than another. Intermediate- to long-term: Little or no difference between behavioural therapy and group exercises for pain or depressive symptoms.
Staal et al. [34]	2008	To determine if injection therapy is more effective than placebo or other treatments for patients with subacute or chronic LBP.		10	Insufficient evidence to support the use of injection therapy in subacute and chronic LBP. Insufficient data to answer whether specific subgroups of patients respond to a specific type of injection therapy.
Deshpande et al. [35]	2007	To determine the efficacy of opioids in adults with CLBP.		4	Quality remark: Although high internal validity scores, the study showed a lack of generalizability, inadequate description of study populations, a poor intention-to-treat analysis, and limited interpretation of functional improvement. The benefits of opioids in clinical practice for the long-term management of CLBP remain questionable.
Dagenais et al. [36]	2007	To determine the efficacy of prolotherapy in adults with CLBP.		5	When used alone, prolotherapy is not an effective treatment for CLBP. When combined with spinal manipulation, exercise, and other co-interventions, prolotherapy may improve CLBP and disability. Quality remark: Conclusions are confounded by clinical heterogeneity amongst studies and by the presence of co-interventions.
Khadilkar et al. [37]	2008	To determine whether TENS is more effective than placebo for the management of CLBP.		4	The current evidence from a small number of placebo-controlled trials does not support the use of TENS in the routine management of CLBP.

CLBP/NSLBP: chronic low back pain/nonspecific low back pain; LBP: low back pain; AC: acupuncture; UC: usual care; TENS: transcutaneous electrical nerve stimulation.

**Table 3:** Characteristics of primary studies included in the systematic reviews.

Systematic review	Year	Patient population (number of studies)	Recruitment and setting(number of studies)	Duration of LBP (years): mean* (range)	Follow-up (months) mean* (range)	Subjects (n) mean* (range)	Age (years) mean* (range)	Outcomes	Data-pooling
Furlan et al.	2005	Chronic SLBP +NSLBP (1), spinal pain syndrome (1), disabling LBP (1), NSLBP (17)	Specialized Unit (13), GP based (2), GP referral (1), Advertisement (3)	8.95 (0.5–12), 12 n.r.	4.15 (13 days–12 months)	74.5 (17–262)	49.67 (38–73)	VAS (9), McGill (2), ODI (2), others (7)	Yes
Urquhart et al.	2008	NSLBP (6), SPLBP+NSLBP (2)	Specialized Unit (3), Advertisement+Specialized Unit (5), GP based (1)	13.94 (3 months–20.3 years)	2.19 (6 weeks–8 months)	65.56 (16–121)	43.9 (26–53)	McGill (1), VAS (2), RMQ (1) others (7)	Yes
Henschke et al.	2010	SP: non-working disabling (1), chronic NSLBP (17), SLBP+NSLBP (1), Quebec taskforce (1), Topographic criteria of Kuorinka et al. (2), hospitalized (2); LBP: employees on sick leave (1), mildly dysfunctional (3), high paraspinal EMG level (1), FAB (1)	Advertisement (4), GP referral (7), GP referral+Advertisement (5), PT (1), Insurance (2), Specialized Unit (11)	7.46 (1.5–12)	12.49 (3 weeks–5 years)	112.19 (20–409)	43.39 (38–50)	NRS (3), McGill (5,) RMQ (4), VAS (2), SF-36 (1), others (12)	Yes
Staal et al.	2008	Chronic SLBP+NSLBP (3), Radiologic severity of Osteoarthritis (Kellgren) (1), NSLBP (6)	Specialized Unit (7), GP setting+Specialized Unit (1), 2 no information (2)	3.29 (1–8.5)	2.32 (after treatment–6 months	84.5 (16–206)	47 (40–65)	VAS (4), Mc Gill (1), RMQ (1), others (4)	No
Deshpande et al.	2007	Chronic NSLBP (4)	3 GP setting+Specialized Unit, 1 Specialized Unit	Only in one reported (6.6)	3.73 (6 weeks–6 months	239 (48–336)	50.3 (42–57)	VAS (2), McGill (2), others (2)	Yes
Dagenais et al.	2007	Chronic NSLBP (3), SPLBP+NSLBP (1), SLBP (1)	GP referral (2), Specialized Unit (1), no information (2)	9.55 (0 days–14 years)	9 (624)	171.4 (74–513)	44.4 (39–50)	VAS (4), McGill (1), RMQ (3), NRS (1)	No
Khadilkar et al.	2008	CLBP: nonspecific (3), acute+chronic LBP (1)	GP-based (1), Advertisement (1), Specialized Unit (1)	3.84 (1.4–6)	1.13 (after treatment–3 months)	133.5 (30–324)	42.88 (31–51)	VAS (3), McGill (1), ODI (1), others (2)	No

LBP: low back pain; SPLBP: specific low back pain; NSLBP: non-specific low back pain; SP: spine pain; EMG: electromyography; GP: general practitioner; includes all primary care-based physician care; Specialized unit: specialist, specialized unit at a hospital; VAS: visual analogue scale; RMQ: Roland Morris Disability Questionnaire; SF-36: Multi-purpose short-form health survey; McGill: McGill Pain Questionnaire; ODI: Oswestry Disability Index; FAB: fear avoidance beliefs.

* Values are calculated as the mean of the mean values reported in the primary trials; the range of the mean values are shown.

### Reporting of Important Prognostic Factors in Primary Studies

**Figure 2:**
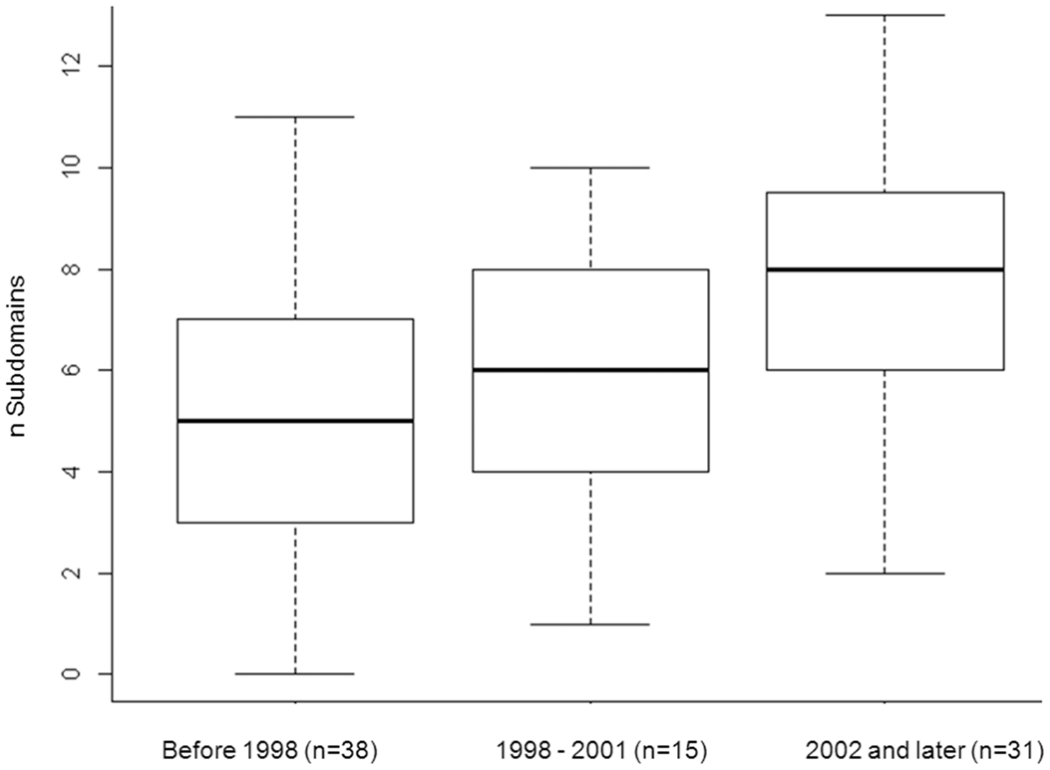
Boxplot showing the number of reported subdomains per primary study over time before and after publication of the CONSORT statement (1996).

The information reported for the domains and subdomains is summarized in Table 4. The data reported most often were data about socio-demographic status and the history of the current LBP. Information about the patient’s general health status, social support, and work-related information was rarely reported. Although statistically significant (p-value = 0.01), the mean number of subdomains with reported information increased after 1998 by fewer than two subdomains (from a mean of 5.4 subdomains to 7.0 subdomains). In studies published after 2001, the median number of subdomains with reported information increased to 8 (of a possible total of 16 subdomains), reflecting a trend towards improved reporting of prognostic important information in recent years (Figure 2).

**Table 4:** Quantity of information in the prognostic subdomains in the 84 RCTs.

Domain	Subdomain	Total	%
General patient characteristics	Sociodemographic information	80	95
	Social support	8	10
Baseline health status	Overall health	22	26
	Overall psychological health	47	56
	Previous LBP	33	39
Work-related factors	Work: psychosocial demands	1	1
	Work: physical demands	6	7
	Work history	39	46
	Work place attributes	3	4
Current LBP	Clinical history	67	80
	Disability related to the complaint	48	57
	Changes related to complaint over time	38	46
Clinical examination findings	Physical examination findings	25	30
	Definition of NSLBP diagnosis	56	67
	Change found during the physical exam	21	25
Interactions with work/society	Compensation issues related to LBP	36	43

LBP: low back pain; NSLBP: non-specific low back pain.

In 17 of the 84 studies (20%), information was reported for all six of the main domains (high SQR). Information was reported for five of the six main domains (moderate SQR) in 30 studies (36%) and for four or fewer domains (low SQR) in 37 studies (44%). The 27 studies investigating cognitive behavioural or educational therapy (termed CBT) provided information for more domains on average (high or moderate SQR for 82%) than studies of other interventions. There was poor reporting in the main domains in studies investigating acupuncture, injection therapy, antidepressants, and opioids (SQR poor in 72–100% of the RCTs) (Table 5).

**Table 5:** Summary of the Score for Quantity of reporting (SQR) types for the RCTs.

	SQR high	SQR moderate	SQR low
All studies (n = 84, 100%)	17 (20%)	30 (36%)	37 (44%)
Acupuncture (n = 18, 21%)	0 (0%)	5 (28%)	13 (72%)
Antidepressants (n = 9, 11%)	1 (5.5%)	1 (5.5%)	8 (89%)
Opioids (n = 4, 5%)	0 (0%)	0 (0%)	4 (100%)
CBT (n = 27, 32%)	11 (41%)	11 (41%)	5 (18%)
TENS: (n = 5, 6%)	1 (20%)	1 (20%)	3 (60%)
EMG (n = 4, 5%)	0 (0%)	2 (50%)	2 (50%)
Reflexology (n = 1, 1%)	0 (0%)	1 (100%)	0 (0%)
Injection Therapy (n = 11, 13%)	2 (18%)	0 (0%)	9 (82%)
Prolotherapy (n = 5, 6%)	2 (40%)	0 (0%)	3 (60%)

CBT: cognitive behavioural therapy or educational therapy; TENS: transcutaneous electrical nerve stimulation; EMG: electromyography; Prolotherapy: Repeated injections of irritant solutions to strengthen lumbosacral ligaments; SQR: Score for quantity of reporting, scoring SQR high: information reported in one or more subdomains for all six main domains; SQR moderate: information reported in one or more subdomains for five main domains; SQR low: information reported in one or more subdomains for four or fewer main domains.

### Comparison of the Classification Systems for Reporting Prognostic Factors Using SQR and the Cochrane Collaboration Guidelines for Baseline Characteristics (CCG-baseline)

In the systematic reviews, the reporting of baseline characteristics was classified as “Can’t tell” in 17 of the 84 studies (20%). The CCG-baseline rating was “Similar” for 59 studies and “Not similar” for 8 studies, indicating that sufficient information for classification was available in most of the studies. The baseline characteristics were classified by the reviewers either as “Similar” or “Not similar” in almost two thirds of the studies with low SQRs (34 studies, 40%) (Table 6). There was thus moderate agreement between the two rating systems, i.e. SQR and CCG-baseline.

**Table 6:** Comparison of the Score for Quantity of Reporting (SQR) categories (high/moderate/low) for the 84 RCTs and the Cochrane Collaboration Guideline-baseline characteristics (CCG-baseline) categories (Similar/Not similar/Can’t tell).

All RCTs (n = 84, 100%)	CCG-baseline: Similar or Not similar (n = 67, 80%)	CCG-baseline: “Can’t tell” (n = 17, 20%)
SQR high (n = 17, 20%)	17 (20%)	0 (0%)
SQR moderate (n = 20, 24%)	16 (19%)	4 (5%)
SQR low (n = 47, 56%)	34 (40%)	13 (15%)

Of the 44 studies pooled for meta-analysis, the SQR was low in 22 studies (50%), and 8 (18%) of the studies were classified as “Can’t tell” according to the CGC-baseline system (Table 7). Five (11%) of the 44 pooled studies were classified as low SQR and “Can’t tell” according to the CGC-baseline system.

**Table 7:** Comparison of the Score for Quantity of Reporting (SQR) categories (high/moderate/low) for the 44 RCTs included in meta-analyses and the Cochrane Collaboration Guideline-baseline characteristics (CCG-baseline) categories (Similar/Not similar/Can’t tell).

All RCTs (n = 44, 100%)	CCG-baseline: Similar or Not similar (n = 36, 81%)	CCG-baseline “Can’t tell” (n = 8, 19%)
SQR high (n = 11, 25%)	9 (20%)	2 (5%)
SQR moderate (n = 11, 25%)	10 (23%)	1 (2%)
SQR low (n = 22, 50%)	17 (39%)	5 (11%)

## Discussion

### Main Findings

In a selection of RCTs that examined various treatments for LBP, there was sparse reporting of relevant baseline characteristics and of prognostic information in particular. This information is needed by clinicians who wish to extrapolate the results to individual patients and by those who conduct meta-analyses who must decide whether it makes sense to pool the results of different studies. The reporting of important prognostic variables could have been more complete in almost half of the assessed trial reports. Even information that could be obtained without great effort and expense, e.g., information on general health status, social support, and work-related conditions, was rarely reported. Half of the studies included in one of the meta-analyses failed to provide enough information for the reader to make an informed decision about whether patients in the primary trials were comparable.

### Comparison with Other Studies

To our knowledge, this is the first study to focus on the reporting of baseline characteristics in RCTs of patients with chronic LBP and to focus on how this issue is addressed in systematic reviews. Baseline patient characteristics are mainly prognostic factors. A comprehensive description of the distribution of these prognostic factors is relevant for determining the applicability of the study results to various patient populations. Comparable analyses have been performed for systematic reviews of prognostic cohort studies [2], [15] and non-randomized intervention studies [16]. The authors of those studies identified incomplete reporting of prognostic factors and recommended a more detailed description of the included patients. Regardless of study design, an incomplete description of patient characteristics increases the risk for bias in interpreting the results of single studies and systematic reviews. A detailed description of the study population helps researchers decide whether it makes sense to pool results from different studies and helps them perform subgroup analyses. The guidelines for conducting systematic reviews mention, without providing detailed instructions, evaluating the comparability of patient populations between primary studies [10], [17], [18], [19]. We found no studies that evaluated the consequences of incomplete reporting of baseline characteristics on the ability of physicians and researchers to interpret RCTs, systematic reviews and meta-analyses.

### Clinical Implications

Many current guideline recommendations are based on the results of systematic reviews and meta-analyses, which are ranked highest in the hierarchy of evidence [20]. A thorough and careful synthesis of primary studies is crucial to warrant this ranking. Critique has been raised on the appraisal of systematic reviews and consequentially on the justification of recommendations in the guidelines. There is a controversy for example about the differences in the rating of the methodological quality of systematic reviews [21]. Another issue of concern physicians repeatedly bring up in educational meetings is the inclusion of RCTs in systematic reviews with conflicting or even contradictory results. Physicians question the comparability of patient populations included in systematic reviews and meta-analyses. Conflicting results in RCTs could reflect a heterogeneous patient population with a range of prognostic profiles [22]. Other explanations for heterogeneity in the results between primary studies on LBP might be, e.g., varying drug dosages or different numbers of training units in exercise therapy, differences in the measurement of the outcome, and outcome measurement at different time points.

While the problem of heterogeneity has been recognized, there is not much research on this issue in conservative treatment for low back pain [23]. However, in clinical practice it is important to know to which degree patient characteristics at baseline affects treatment efficacy. From a clinician’s perspective it seems reasonable to assume that certain treatments (e.g., cognitive behavioral therapy) are more effective in patients presenting with yellow flags (e.g., fear avoidance beliefs, distress) or depression. It is therefore relevant to know about specific treatment effects in subgroups of patients. Clinicians expect from researchers that this heterogeneity in treatment effects is scrutinized and relevant prognostic patient characteristics are considered in the synthesis of RCTs in order to offer an evidence-based and goal oriented health care to the patients [24].

Various concerns on the value of systematic reviews and meta-analyses have been raised in the past years that are beyond the scope of this analysis [20], [25], [26], [27], [28]. Systematic reviews offer the possibility to exploit the heterogeneity of prognostic profiles and to conduct subgroup analyses. Thus, reporting the relevant baseline characteristics in primary studies is critical for the quality of the systematic review. Further, collaboration between clinicians and methodologists allows for a meaningful pooling of data in meta-analyses and to examine treatment effects in different groups of patients with LBP. A striking example that underlines the importance of clinical knowledge and subgroup analyses is a recent systematic review investigating vitamin D supplementation for the protection of hip fracture [29]. While the overall effect in this systematic review including all patients, irrespective of their vitamin D blood level at baseline, was not different to placebo, vitamin D protected against hip fractures in individuals with low vitamin D levels at the time of inclusion in the trials.

### Limitations of the Study

While we applied robust methodology and a systematic approach to assess the reporting of prognostic information in RCTs, the current study has some limitations. In using a score based on domains, all prognostic information was given equal weight. We are aware that this may be an over-simplification. The cut-off for ‘low SQR’ used in the current study was chosen arbitrarily, and more studies would have fulfilled the quality criteria if the cut-off was lower. Because we accepted that any reported information was sufficient to fulfil a domain, we think the cut-off we used was reasonable. Accordingly, non-reporting of information in two or more main domains represents a risk for misinterpretation of results not only in primary studies but also in systematic reviews. We support current efforts to standardize measurements of prognostic factors and reporting in back pain research that will make it easier to compare studies in the future [3], [30].

Another limitation of our study is the focus was only on RCTs that were included in systematic reviews published in the Cochrane library. Inclusion of RCTs in systematic reviews published in other databases might give different results. We chose the Cochrane systematic reviews as they are widely recognized as setting the standard for the evaluation of healthcare interventions [31]. Furthermore, the standardized risk of bias assessment in all systematic reviews ensures similar assessment in the different reviews. While the Cochrane reviews are relatively recent, the most recent trial included in our analysis was published in 2009, and more important prognostic information might be reported in more recent studies. Our analysis of changes in reporting over time showed a small but statistically significant increase in reporting in the ten years after publication of the CONSORT statements.

### Conclusion

Missing prognostic information potentially threatens the external validity (i.e. generalizability or applicability) not only of primary studies but also of systematic reviews that evaluate treatments for LBP. A detailed description of baseline characteristics, including important prognostic information, will help clinicians and researchers make informed decisions about whether the results of a study or a systematic review apply to their patients.

## References

[1] Schulz KF, Altman DG, Moher D (2010) Group C (2010) CONSORT 2010 statement: updated guidelines for reporting parallel group randomized trials. Ann Intern Med 152: 726–732. 20335313 10.7326/0003-4819-152-11-201006010-00232

[2] Hayden JA, Chou R, Hogg Johnson S, Bombardier C (2009) Systematic reviews of low back pain prognosis had variable methods and results: guidance for future prognosis reviews. J Clin Epidemiol 62 781–796: e781. 10.1016/j.jclinepi.2008.09.004 19136234

[3] Pincus T, Santos R, Breen A, Burton AK, Underwood M, et al (2008) A review and proposal for a core set of factors for prospective cohorts in low back pain: a consensus statement. Arthritis Rheum 59: 14–24. 18163411 10.1002/art.23251

[4] Nour K, Laforest S, Gauvin L, Gignac M (2006) Behavior change following a self-management intervention for housebound older adults with arthritis: an experimental study. International Journal of Behavioral Nutrition and Physical Activity 3: 12. 16734904 10.1186/1479-5868-3-12 PMC1525193

[5] Laupacis A, Wells G, Richardson W (1994) Users' Guides to the Medical Literature: V. how to use an article about prognosis. JAMA: The Journal of the American Medical Association 272: 234–237. 8022043 10.1001/jama.272.3.234

[6] Balagu F, Mannion A, Pellis F, Cedraschi C (2012) Non-specific low back pain. Lancet 379: 482–491. 21982256 10.1016/S0140-6736(11)60610-7

[7] Hayden JA, Dunn KM, van der Windt DA, Shaw WS (2010) What is the prognosis of back pain? Best Practice & Research Clinical Rheumatology 24: 167–179. 20227639 10.1016/j.berh.2009.12.005

[8] van Tulder MW, Assendelft WJ, Koes BW, Bouter LM (1997) Method guidelines for systematic reviews in the Cochrane Collaboration Back Review Group for Spinal Disorders. Spine (Philadelphia, Pa 1976) 22: 2323–2330. 10.1097/00007632-199710150-00001 9355211

[9] van Tulder M, Furlan A, Bombardier C, Bouter L, Editorial Board of the Cochrane Collaboration Back Review G (2003) Updated method guidelines for systematic reviews in the cochrane collaboration back review group. Spine (Phila Pa 1976) 28: 1290–1299. 12811274 10.1097/01.BRS.0000065484.95996.AF

[10] Furlan AD, Pennick V, Bombardier C, van Tulder M, Editorial Board CBRG (2009) 2009 updated method guidelines for systematic reviews in the Cochrane Back Review Group. Spine (Phila Pa 1976) 34: 1929–1941. 19680101 10.1097/BRS.0b013e3181b1c99f

[11] Liberati A, Altman DG, Tetzlaff J, Mulrow C, Gøtzsche PC, et al.. (2009) The PRISMA statement for reporting systematic reviews and meta-analyses of studies that evaluate healthcare interventions: explanation and elaboration. BMJ 339. 10.1136/bmj.b2700 PMC2714672 19622552

[12] Manchikanti L, Singh V, Cash KA, Pampati V, Datta S (2009) A comparative effectiveness evaluation of percutaneous adhesiolysis and epidural steroid injections in managing lumbar post surgery syndrome: a randomized, equivalence controlled trial. Pain Physician 12: E355–368. 19935992

[13] Trigkilidas D (2010) Acupuncture therapy for chronic lower back pain: a systematic review. Ann R Coll Surg Engl 92: 595–598. 20529520 10.1308/003588410X12699663904196 PMC3229352

[14] Henschke N, Ostelo RW, van Tulder MW, Vlaeyen JW, Morley S, et al.. (2010) Behavioural treatment for chronic low-back pain. Cochrane Database Syst Rev: CD002014. 10.1002/14651858.CD002014.pub3 PMC7065591 20614428

[15] Hayden JA, Côté P, Bombardier C (2006) Evaluation of the Quality of Prognosis Studies in Systematic Reviews. Annals of Internal Medicine 144: 427–437. 16549855 10.7326/0003-4819-144-6-200603210-00010

[16] Deeks JJ, Dinnes J, D'Amico R, Sowden AJ, Sakarovitch C, et al.. (2003) Evaluating non-randomised intervention studies. Health Technol Assess 7: iii–x, 1–173. 10.3310/hta7270 14499048

[17] Research CoSfSRoCE, Medicine Io (2011) Finding What Works in Health Care: Standards for Systematic Reviews; Eden J, Levit L, Berg A, Morton S, editors: The National Academies Press. 24983062

[18] Higgins JPT, Green S (2011) Cochrane Handbook for Systematic Reviews of Interventions Version 5.1.0 The Cochrane Collaboration.

[19] Dissemination CfRa (2009) CRD's guidance for undertaking reviews in health care. Third Edition ed: CRD, University of York.

[20] Egger M, Ebrahim S, Smith GD (2002) Where now for meta-analysis? Int J Epidemiol 31: 1–5. 11914281 10.1093/ije/31.1.1

[21] Chou R, Atlas SJ, Loeser JD, Rosenquist RW, Stanos SP (2011) Guideline Warfare Over Interventional Therapies for Low Back Pain: Can We Raise the Level of Discourse? The Journal of Pain 12: 833–839. 21742563 10.1016/j.jpain.2011.04.012

[22] Jane wit D, Horwitz R, Concato J (2010) Variation in results from randomized, controlled trials: stochastic or systematic? J Clin Epidemiol 63: 56–63. 19740624 10.1016/j.jclinepi.2009.02.010

[23] Fourney D, Andersson G, Arnold P, Dettori J, Cahana A, et al (2011) Chronic low back pain: a heterogeneous condition with challenges for an evidence-based approach. Spine (Philadelphia, Pa 1976) 36: S1–S9. 10.1097/BRS.0b013e31822f0a0d 21952181

[24] Ostelo R, Croft P, van der Weijden T, van Tulder M (2010) Challenges in using evidence to inform your clinical practice in low back pain. Best Practice & Research Clinical Rheumatology 24: 281–289. 20227648 10.1016/j.berh.2009.12.006

[25] Freeman MD (2010) Clinical practice guidelines versus systematic reviews; which serve as the best basis for evidence-based spine medicine? The Spine Journal 10: 512–513. 20494813 10.1016/j.spinee.2010.04.006

[26] Willis B, Quigley M (2011) The assessment of the quality of reporting of meta-analyses in diagnostic research: a systematic review. BMC Medical Research Methodology 11: 163. 22151233 10.1186/1471-2288-11-163 PMC3258221

[27] Pambrun E, Bouteloup V, Thibaut R, Asselineau J, de-Ldinghen V, et al (2010) On the validity of meta-analyses: exhaustivity must be warranted, exclusion of duplicate patients too. J Clin Epidemiol 63: 342–343. 20079606 10.1016/j.jclinepi.2009.07.021

[28] Kirkham JJ, Dwan KM, Altman DG, Gamble C, Dodd S, et al.. (2010) The impact of outcome reporting bias in randomised controlled trials on a cohort of systematic reviews. BMJ 340. 10.1136/bmj.c365 20156912

[29] Bischoff-Ferrari HA, Willett WC, Orav EJ, Lips P, Meunier PJ, et al (2012) A pooled analysis of vitamin D dose requirements for fracture prevention. N Engl J Med 367: 40–49. 22762317 10.1056/NEJMoa1109617

[30] Dionne C, Dunn K, Croft P, Nachemson A, Buchbinder R, et al (2008) A consensus approach toward the standardization of back pain definitions for use in prevalence studies. Spine (Philadelphia, Pa 1976) 33: 95–103. 10.1097/BRS.0b013e31815e7f94 18165754

[31] Tricco A, Tetzlaff J, Pham B, Brehaut J, Moher D (2009) Non-Cochrane vs. Cochrane reviews were twice as likely to have positive conclusion statements: cross-sectional study. J Clin Epidemiol 62 380–386: e381. 10.1016/j.jclinepi.2008.08.008 19128940

[32] Furlan AD, van Tulder MW, Cherkin DC, Tsukayama H, Lao L, et al.. (2005) Acupuncture and dry-needling for low back pain. Cochrane Database Syst Rev: CD001351. 10.1002/14651858.CD001351.pub2 PMC12145953 15674876

[33] Urquhart DM, Hoving JL, Assendelft WW, Roland M, van Tulder MW (2008) Antidepressants for non-specific low back pain. Cochrane Database Syst Rev: CD001703. 10.1002/14651858.CD001703.pub3 PMC7025781 18253994

[34] Staal JB, de Bie R, de Vet HC, Hildebrandt J, Nelemans P (2008) Injection therapy for subacute and chronic low-back pain. Cochrane Database Syst Rev: CD001824. 10.1002/14651858.CD001824.pub3 PMC7096223 18646078

[35] Deshpande A, Furlan A, Mailis-Gagnon A, Atlas S, Turk D (2007) Opioids for chronic low-back pain. Cochrane Database Syst Rev: CD004959. 10.1002/14651858.CD004959.pub3 17636781

[36] Dagenais S, Yelland MJ, Del Mar C, Schoene ML (2007) Prolotherapy injections for chronic low-back pain. Cochrane Database Syst Rev: CD004059. 10.1002/14651858.CD004059.pub3 PMC6986690 17443537

[37] Khadilkar A, Odebiyi DO, Brosseau L, Wells GA (2008) Transcutaneous electrical nerve stimulation (TENS) versus placebo for chronic low-back pain. Cochrane Database Syst Rev: CD003008. 10.1002/14651858.CD003008.pub3 PMC7138213 18843638

